# Ultrafast visual detection of *Streptococcus suis* via HtpsC‐targeted LAMP–CRISPR–Cas12a coupled with lateral flow immunochromatography

**DOI:** 10.1002/2211-5463.70353

**Published:** 2026-10-16

**Authors:** Liu‐xin Li, Miao Xin, Xiao‐wen Cui, Shi‐yin Yu, Fei Meng, Min Cao, Xiao‐hui Xiong, Yi‐chen Lu

**Affiliations:** ^1^ College of Food Science and Light Industry Nanjing Tech University 30 South Puzhu Road Nanjing 211816 Jiangsu Province China; ^2^ School of Excellence in Biomedical Engineering China Pharmaceutical University Nanjing 211198 China

**Keywords:** CRISPR–Cas12a, detection, LAMP, LFIA, *Streptococcus suis*

## Abstract

This study describes a rapid, visual detection platform for *Streptococcus suis*, a significant zoonotic pathogen, by integrating loop‐mediated isothermal amplification (LAMP) with CRISPR–Cas12a and a lateral flow immunochromatographic strip (LFIA) readout. Targeting the conserved HtpsC gene, specific LAMP primers and a PAM‐compliant CrRNA were designed. Following isothermal amplification, the Cas12a/CrRNA complex recognizes the HtpsC amplicon, initiating target‐specific cleavage and subsequent trans‐cleavage of a dual‐labeled ssDNA reporter (FAM‐BIO), enabling visual detection on a LFIA; a design which reduces false positives. The optimized assay detected HtpsC‐positive *S. suis* strains within 45 min and achieved a visual detection limit of 1.1 × 10^2^ CFU/mL under laboratory conditions, with no cross‐reactivity observed against the tested nontarget bacteria. In addition to pure‐culture dilutions, the assay was further evaluated using spiked porcine blood samples and achieved similar detection performance, supporting its preliminary applicability in a complex biological matrix. Overall, this study provides an HtpsC‐targeted visual detection workflow for screening of *S. suis* and is expected to be further applied to on‐site rapid detection of clinical samples.

## Graphical abstract

A rapid visual test combines DNA amplification, CRISPR–based target verification, and a lateral flow strip to detect *Streptococcus suis*. The assay gives results in about 45 min, reduces false‐positive interpretation, and does not require fluorescence equipment.

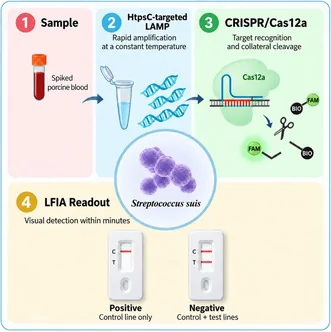

**Abbreviations**
BIO: Biotin
Bst: Bacillus stearothermophilus DNA polymerase
Cas12a: CRISPR–associated protein 12/also called Cpf1(Cutting PAM‐specific Endonuclease from Prevotella and Francisella 1)
C line: Control line
CRISPR: Clustered regularly spaced short palindromic repeats
CrRNA: Clustered regularly interspaced short palindromic repeats RNA
FAM: 6‐Carboxyfluorescein
HtpsC: Type II histidine triad protein C
LAMP: Loop‐mediated isothermal amplification
LFIA: Lateral flow immunoassay
PAM: Protospacer adjacent motif
PCR: Polymerase chain reaction
ssDNA: Single‐stranded DNA
*S. suis*
: *Streptococcus suis*
T line: Test line

*Streptococcus suis* is an important Gram‐positive zoonotic pathogen that can be transmitted to humans through close contact with infected pigs or consumption of contaminated pork products [1]. *S. suis* can cause severe infections, including meningitis, sepsis, endocarditis, and acute death [2]. Since the earliest reported human cases in Denmark in 1968, *S. suis* infections have been reported worldwide, with more than 1500 cases documented in 34 countries between 2002 and 2013 [3, 4]. In addition to pigs, *S. suis* has also been reported in other animals, including cats, dogs, horses, and poultry [5]. To date, *S. suis* has been classified into 29 serotypes based on capsular polysaccharide antigenicity, among which Serotypes 1, 2, 1/2, 7, 9, and 14 are considered pathogenic, with Serotype 2 being the most virulent and frequently detected in clinical cases [6]. Given its diversity, wide distribution, high pathogenicity, and multiple transmission routes, *S. suis* poses a serious threat to the swine industry and public health, making timely and accurate surveillance essential for infection control and public health monitoring [1].

The type II histidine triad protein C (HtpsC), a surface protein of *Streptococcus suis*, derives its name from the presence of four to six conserved histidine trimer domains (HxxHxH) in its amino acid sequence. Experimental evidence confirms HtpsC as a novel adhesin in *S. suis* Serotype 2, contributing to the pathogenesis of the highly virulent strain 05ZYH33 [7]. This protein is distributed across multiple *S. suis* serotypes and represents a crucial virulence factor [8]. It is noteworthy that HtpsC displays a marked degree of conservation in its histidine trimer gene sequences, particularly within the N‐terminal region of each protein. Given the exceptional conservation of these sequences and their established role in *S. suis* pathogenicity, they serve as excellent molecular targets for bacterial detection [9].

Currently, the diagnosis of *Streptococcus suis* infection relies on immunological testing and nucleic acid testing [10, 11, 12]. Traditional testing methods are time‐consuming, labor‐intensive, and relatively insensitive [13, 14]. Recent advances in molecular technology have led to the development of new, more sensitive methods for detecting *Streptococcus suis*, such as PCR and qPCR, but these methods have high requirements for the operating environment, equipment, and personnel [15, 16]. Enzyme‐linked immunosorbent assay (ELISA) and agglutination tests are easy to perform and provide rapid results, but their disadvantages include low sensitivity, high material costs, and complex and time‐consuming procedures [17, 18, 19]. Currently, compared with traditional detection technologies, such as ELISA and PCR, loop‐mediated isothermal amplification (LAMP) technology has attracted considerable attention from researchers in many fields due to its short reaction time, high sensitivity, good specificity, ease of visualization, and suitability for point‐of‐care testing. Loop‐mediated isothermal amplification is a rapid nucleic acid amplification technique performed under isothermal conditions. It involves designing four to six specific primers for the target gene and utilizing the strand displacement activity of Bst enzyme to react at 60–65 °C for 30–60 min, generating magnesium pyrophosphate precipitation to achieve rapid and efficient amplification of the target gene [20, 21, 22]. However, LAMP reactions often yield false‐positive results due to environmental contamination, complex sample components, nonspecific amplification, or primer dimerization [23, 24]. Once false‐positive results occur, the reaction must be repeated, reagents replaced, and the amplification system redesigned [6, 25, 26, 27, 28]. In recent years, LAMP technology has been widely applied in fields, such as pathogen and food detection, but false‐positive results often occur due to improper operation or aerosol contamination.

The clustered regularly interspaced short palindromic repeats (CRISPR)–associated (Cas) system is a powerful genome‐editing platform. Originally identified as an adaptive immune mechanism in prokaryotes, these systems have revolutionized biotechnology since they were developed as gene‐editing tools in 2012 [29]. Among these systems, the type V Class II nuclease Cas12a (formerly Cpf1) has emerged as a transformative technology in molecular diagnostics due to its unique enzymatic properties [29, 30, 31]. Unlike Cas9, Cas12a's structural variations confer distinct catalytic activities. When guided by a CrRNA, Cas12a mediates both cis‐cleavage of target double‐stranded DNA and promiscuous trans‐cleavage of single‐stranded DNA (ssDNA). This latter feature has been repurposed as a highly sensitive signal amplification strategy. CRISPR–Cas systems are classified into two major classes (Class 1 and Class 2) and six types (I–VI) based on the composition of their effector proteins. The Type V system, to which Cas12a belongs, has gained prominence in pathogen detection due to its operational simplicity and broad targeting scope, recognizing T‐rich PAM sequences [32, 33]. Recent studies have demonstrated the successful integration of LAMP–CRISPR/Cas12a platforms for detecting foodborne pathogens, including *Escherichia coli*, *Salmonella spp*., *Vibrio parahaemolyticus*, and *Pseudomonas aeruginosa* [34, 35, 36, 37]. Research has found that combining it with isothermal amplification technologies, such as LAMP, further improves detection specificity while effectively correcting the false positives commonly found in LAMP testing. This combination offers promising prospects for rapid, on‐site testing [38].

Lateral flow immunoassay (LFIA) has become the gold standard for point‐of‐care visual testing, utilizing antibody‐nucleic acid conjugate interactions to generate a colorimetric signal on a nitrocellulose membrane. Recent advances have combined LFIA with nucleic acid amplification technology, in which biotin‐FAM‐labeled amplicons are captured by anti‐FAM antibodies to generate a visible test strip [39, 40]. Notably, the CRISPR–Cas system can regulate signal generation on the lateral flow strip by activating Cas12a to cleave reporter molecules (e.g., FAM‐biotin ssDNA) through its nuclease activity, thereby expanding the application of LFIA as a visualization tool [41]. This integration has been successfully applied to a *Salmonella* detection system that combines the rapid amplification of LAMP with the specificity of Cas12a, enabling visualization of results within 40 min using LFIA (detection limit: 10 CFU/mL) [35].

In this study, we developed an HtpsC‐targeted LAMP–CRISPR/Cas12a–LFIA workflow for visual detection of *S. suis* and used CRISPR/Cas12a‐mediated sequence confirmation to reduce false‐positive interpretation associated with nonspecific LAMP amplification.

## Materials and methods

### Experimental strains and culture conditions

Strain *Streptococcus suis 2 (05ZYH33)* was isolated from a patient with toxic shock syndrome in Ziyang, Sichuan Province, in 2005. *S. suis 1 (5428)*, *S. suis 2 (NCTC 10234)*, *S. suis 4 (2524)*, *S. suis 7 (8074)*, *S. suis 9 (22083)*, and *S. suis 14 (13730)* were kindly provided by Professor Marcelo Gottschalk from Canada. *S. suis 2 (T15)* originated from the Greef Laboratory in the Netherlands and was provided by Professor Astrid de Greeff. *S. suis 2 (WZ48‐1)* was provided by Professor Li Ming from the Army Medical University. *E. coli (GDMCC 801268)*, *S. enteriditis (ATCC 14028)*, *S. aureus (ATCC 29213)*, and *L. monocytogenes (ATCC 54004)* were kindly provided by the Nanjing Station of the National Light Industry Food Quality Supervision and Testing Center. *S. pyogenes (ATCC 19615)*, *S. agalactiae (GDMCC 1408)*, and *E. faecalis (ATCC 51299)* were purchased from the Guangdong Provincial Microbial Culture Collection Center. Detailed strain information is provided in Table S1.

Before experimental procedures, *Streptococcus suis* strains were initially activated through overnight incubation at 37 °C on Todd‐Hewitt Broth (THB) agar plates. Subsequently, individual colonies were aseptically transferred into 5 mL of THB broth for secondary activation, maintaining the same incubation conditions (37 °C, overnight). Both primary and secondary activation processes were consistently performed using THB‐based media, with solid and liquid formulations employed for the respective activation stages.

### Principal reagents and equipment

Bst DNA polymerase 2.0 was purchased from Novazenz Biotechnology Co., Ltd. (Nanjing, China). The 6His‐MBP‐TEV‐huLbCpf1 plasmid was obtained from Wuhan Miaoling Biotechnology Co., Ltd. (Wuhan, China). LbCpf1 (Cas12a) protein was expressed and purified in our laboratory as described in the Supporting Information. dNTP Mix was purchased from Shengrui Biotechnology Co., Ltd. (Beijing, China). SYBR Green I was purchased from Solarbio Science & Technology Co., Ltd. (Beijing, China). LAMP primers, PCR primers, ssDNA reporter probes, and CrRNAs were synthesized by General Biosystems Co., Ltd. (Suzhou, China). Anti‐FAM and anti‐biotin antibodies for LFIA strip preparation were obtained from Sangon Biotech Co., Ltd. (Shanghai, China).

Fluorescence signals were collected using a Gentier 96E real‐time PCR analyzer (Xi'an Tianlong Science and Technology Co., Ltd., Xi'an, China). Membrane spraying and strip assembly were carried out using an HGS510 membrane spraying instrument (Hangzhou Fenghang Technology Co., Ltd., Hangzhou, China). Routine centrifugation was performed using an S1010 benchtop centrifuge (Scilogex, Rocky Hill, CT, USA). Gel electrophoresis was conducted using a JY‐300C electrophoresis apparatus (Beijing Junyi Dongfang Electrophoresis Equipment Co., Ltd., Beijing, China), and gel images were acquired using a Tanon‐1650 gel imaging system (Xi'an Tianlong Science and Technology Co., Ltd., Xi'an, China).

### LAMP primer design and screening

The conserved and specific HtpsC gene in *Streptococcus suis* was selected as the target gene. LAMP primers were initially designed using PrimerExplorer (https://primerexplorer.jp/e/). Candidate primer sets were further evaluated using the Oligo software for melting temperature, GC content, self‐dimer formation, cross‐dimer formation, and hairpin structure. The final primer set selected for the HtpsC‐targeted LAMP assay is listed in Table S2.

### Optimization of the LAMP reaction system

For template preparation, bacterial cells were collected by centrifugation at 10 000× ***g*** for 10 min and resuspended in sterile nuclease‐free water. The bacterial concentration was determined by the standard plate count method following serial dilution and spread plating, and 10‐fold gradient dilutions were prepared as required for subsequent experiments. Prior to the LAMP reaction, the bacterial suspensions were subjected to boiling lysis at 100 °C for 10 min, followed by brief centrifugation. The resulting lysate was used as the amplification template. Unless otherwise indicated, 1 μL of the processed bacterial suspension was added to each 25 μL LAMP reaction.

### Construction and optimization of the LAMP–CRISPR/Cas12a detection platform

A LAMP–CRISPR/Cas12a workflow was established by combining LAMP amplicons with a pre‐assembled Cas12a/CrRNA ribonucleoprotein complex. The HtpsC target region was selected based on the presence of a compatible 5′‐TTTV‐3′ PAM sequence. Four candidate CrRNAs were designed using an online sgRNA design tool and synthesized for screening; their sequences are listed in Table S2.

A dual‐labeled ssDNA reporter (5′‐BIO‐TTTTTATTTTT‐FAM‐3′) was used for Cas12a‐mediated trans‐cleavage detection. The initial Cas12a reaction was performed in a 20 μL system containing reaction buffer, purified target amplicon, ssDNA reporter, CrRNA, Cas12a protein, and nuclease‐free water. The CrRNA identity, reaction volume, Cas12a: CrRNA molar ratio, incubation time, and ssDNA reporter concentration were subsequently optimized.

For CrRNA screening and cis‐cleavage validation, a 1323‐bp HtpsC fragment was amplified using gene‐specific PCR primers listed in Table S2 and used as the cleavage substrate. After Cas12a/CrRNA‐mediated cleavage, the products were analyzed by agarose gel electrophoresis. The optimized CRISPR/Cas12a conditions were then applied to LAMP product verification and LFIA‐based detection.

### Development of a combined detection method using LAMP–CRISPR/Cas12a and lateral flow immunoassay test strips

We employed in‐house fabricated LFIA strips for instrument‐free visual readout. Each strip measured 4 × 60 mm and was assembled on a backing card with a sample pad, conjugate pad, nitrocellulose membrane, and absorbent pad (Fig. 1A). Adjacent components were overlapped by 2 mm to ensure continuous capillary flow. Colloidal gold nanoparticles with a diameter of 15 nm were conjugated with anti‐biotin antibodies and preloaded onto the conjugate pad. Anti‐FAM antibody and goat anti‐rabbit antibody were immobilized on the nitrocellulose membrane as the test line (T line) and control line (C line), respectively. For strip preparation, 10 μg of 1 mg/mL antibody solution was used for coating each line.

**Fig. 1:**
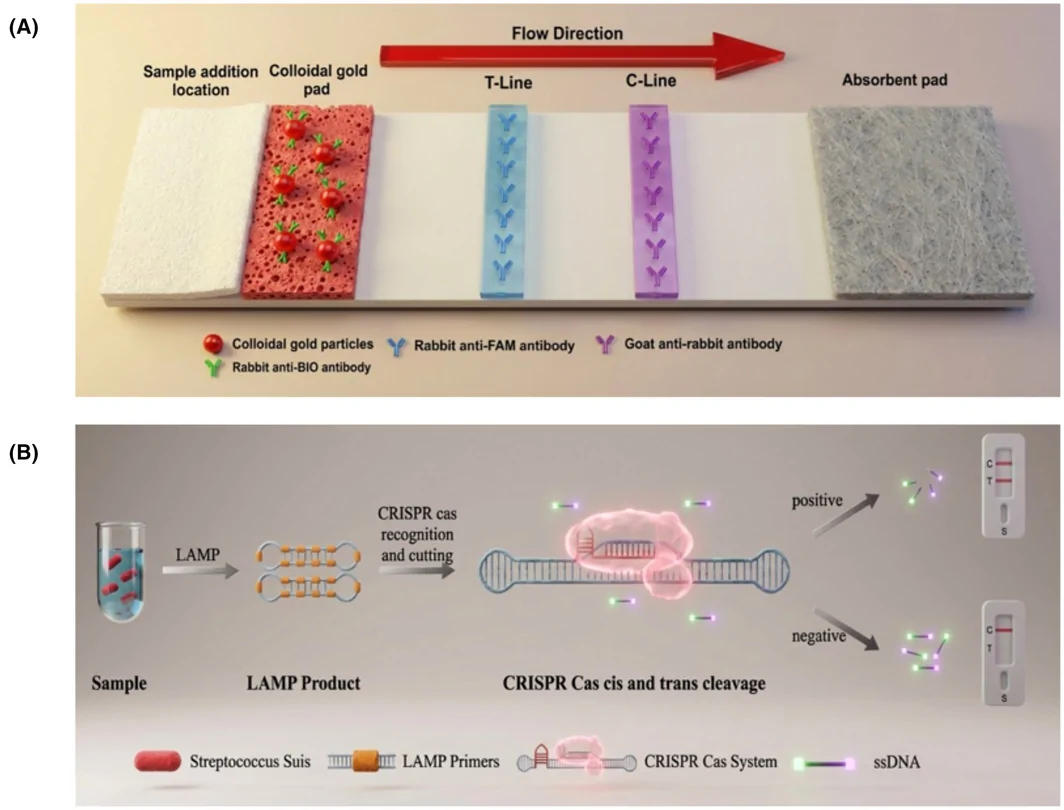
(A) Schematic illustration of the LFIA strip structure. The strip consists of a sample pad, conjugate pad, nitrocellulose membrane, and absorbent pad assembled on a backing card. Anti‐FAM antibody and goat anti‐rabbit antibody were immobilized on the nitrocellulose membrane as the test line (T line) and control line (C line), respectively. (B) Schematic illustration of the HtpsC‐targeted LAMP–CRISPR/Cas12a–LFIA detection workflow for *Streptococcus suis*.

After LAMP amplification, the products were introduced into the CRISPR/Cas12a reaction for target recognition and reporter cleavage. The reaction mixture was diluted to 100 μL with nuclease‐free water and applied to the LFIA sample pad. Visual bands appeared within approximately 2 min (Fig. 1B). The ssDNA reporter was dual‐labeled with biotin (BIO) and fluorescein (FAM), allowing LFIA‐based interpretation according to its cleavage state.

The LFIA readout follows an inverse T‐line interpretation. In negative samples, Cas12a is not activated and the intact BIO–FAM reporter is captured at the T line through anti‐FAM antibody while recruiting colloidal gold–anti‐BIO conjugates, resulting in visible T and C lines. In positive samples, target‐activated Cas12a cleaves the reporter, preventing formation of the gold‐bearing complex at the T line; therefore, only the C line remains visible. Thus, suppression or disappearance of the T line with a visible C line is interpreted as positive, whereas the presence of both T and C lines is interpreted as negative. A visible C line is required for test validity.

### Analytical sensitivity and specificity of the LAMP–CRISPR/Cas12a–LFIA assay

*S. suis* 2 (05ZYH33) was prepared as a 10‐fold serial dilution series ranging from 1 × 10^0^ to 1 × 10^7^ CFU/mL to determine the analytical sensitivity of the assay. For each dilution, aliquots were subjected to LAMP amplification under the optimized conditions, followed by Cas12a–CrRNA cleavage and LFIA readout using the dual‐labeled BIO–FAM reporter.

Analytical specificity was assessed using the strain panel listed in Table S1, which included multiple *S. suis* strains representing different serotypes and several non‐*S. suis* bacterial species. For each organism, genomic templates were processed through the same workflow, including LAMP amplification, Cas12a cleavage, and LFIA readout.

### Validation in spiked blood samples

Fresh porcine blood, purchased from a local market and obtained from pigs slaughtered for food production, was used to evaluate the applicability of the proposed method in a complex biological matrix. Known concentrations of S. suis 2 (05ZYH33) were artificially introduced into the blood samples, followed by the same LAMP–CRISPR/Cas12a–LFIA detection procedure described above. Nonspiked porcine blood samples were included as negative controls to assess potential background interference. As no animals were specifically handled, sampled, or euthanized for this study, no animal ethics approval was required.

### Conventional PCR reaction

We compared the PCR assay commonly performed at primary‐level laboratories with the method developed in this study. For conventional PCR amplification, 2× PrimeSTAR Max Premix (Vazyme Biotech, Nanjing, China) was used according to the manufacturer's instructions. Each 25 μL PCR reaction contained 1 μL of template DNA, 1 μL of each primer, 12.5 μL of 2× PrimeSTAR Max Premix, and deionized water to a final volume of 25 μL. The PCR amplification was performed under the following cycling conditions: initial denaturation at 95 °C for 3 min, followed by 30 cycles of denaturation at 95°C for 10 s, annealing at 60 °C for 10 s, and extension at 72 °C for 5 s. The PCR products were subsequently analyzed by agarose gel electrophoresis, and the resulting bands were visualized for further analysis.

### Statistical analysis

All experiments were performed in triplicate unless otherwise stated. For qualitative LFIA results, assay reproducibility was evaluated based on the consistency of visual interpretation across three independent replicates. The limit of detection (LOD) was defined as the lowest bacterial concentration that consistently produced the expected positive LFIA pattern in all replicates.

## Results

### Optimization of the LAMP amplification conditions

To optimize the HtpsC‐targeted LAMP assay, reaction temperature and key reagent concentrations were evaluated under otherwise identical conditions. Reaction temperatures of 61 °C, 63 °C, 65 °C, 67 °C, and 69 °C were first tested. As shown in Fig. 2A, amplification was observed at all tested temperatures, but 65 °C produced the earliest threshold time (Tt) and the highest endpoint fluorescence signal. Therefore, 65 °C was selected as the optimal reaction temperature. Under this condition, the fluorescence signal reached a plateau within approximately 30 min; thus, an incubation time of 30 min followed by enzyme inactivation at 85 °C for 5 min was used for subsequent experiments.

**Fig. 2:**
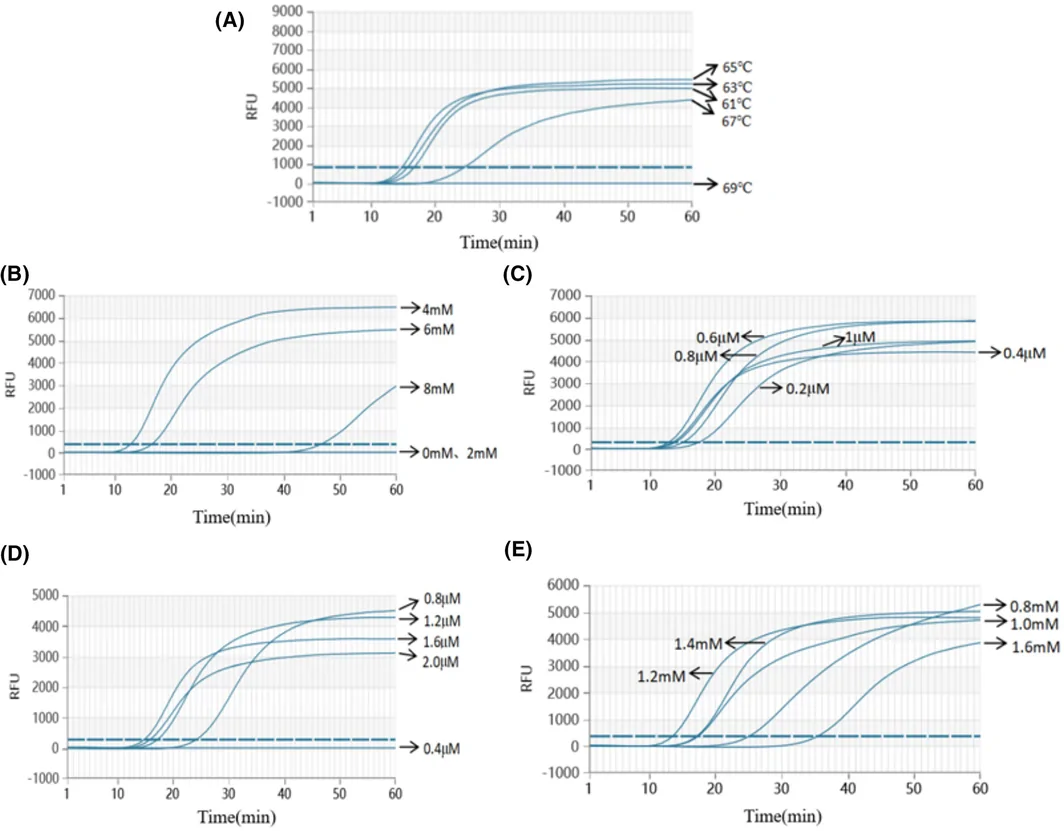
Optimization of HtpsC‐targeted LAMP reaction conditions. (A) Optimization of reaction temperature. (B) Optimization of Mg^2+^ concentration. (C) Optimization of outer primer concentration. (D) Optimization of inner primer concentration. (E) Optimization of dNTP concentration.

The concentrations of Mg^2+^, primers, and dNTPs were then optimized stepwise at 65 °C. Mg^2+^ concentrations of 0, 2, 4, and 6 mm were tested, and 4 mm generated the strongest and fastest amplification signal (Fig. 2B). For the outer primers F3/B3, concentrations ranging from 0.2 to 1.0 μm were evaluated, and 0.6 μm provided the best amplification performance (Fig. 2C). For the inner primers FIP/BIP, concentrations ranging from 0.4 to 2.0 μm were tested, and 1.2 μm produced the shortest Tt and strongest endpoint fluorescence (Fig. 2D). dNTP concentrations of 0.8, 1.0, 1.2, 1.4, and 1.6 mm were further evaluated, with 1.2 mm yielding the optimal amplification efficiency (Fig. 2E).

Based on these results, the optimized LAMP reaction was performed in a 25 μL system containing 2.5 μL of 10× ThermoPol Buffer, 4 mm Mg^2+^, 1.2 mm dNTPs, 0.6 μm each outer primer, 1.2 μm each inner primer, 1 μL of Bst DNA polymerase, 1 μL of SYBR Green I, and 1 μL of template bacterial suspension. The reaction was incubated at 65 °C for 30 min and then terminated at 85°C for 5 min. These optimized conditions were used for subsequent CRISPR/Cas12a detection and LFIA readout.

### Sequence optimization results for CrRNA

The relationship between the four designed CrRNAs, LAMP primers, and the HtpsC target gene is illustrated in Fig. S1. Under identical experimental conditions, the cleavage efficiency of the four different CrRNAs was validated, and the results of agarose gel electrophoresis are shown in Fig. 3A‐a. In the cleavage system containing CrRNA‐2, the 1323‐bp PCR product was completely cleaved into two fragments of approximately 900 bp and 400 bp, as observed in Lane 2 of Fig. 3A‐a. In contrast, the cleavage systems containing CrRNA‐1, CrRNA‐3, and CrRNA‐4, partial cleavage of the 1323‐bp PCR product was observed, as indicated in Lanes 1, 3, and 4 of Fig. 3A‐a. In summary, the CRISPR–Cas system demonstrated the highest target gene cleavage efficiency when CrRNA‐2 was utilized. Therefore, CrRNA‐2 was identified as the optimal CrRNA for the CRISPR–Cas system.

**Fig. 3:**
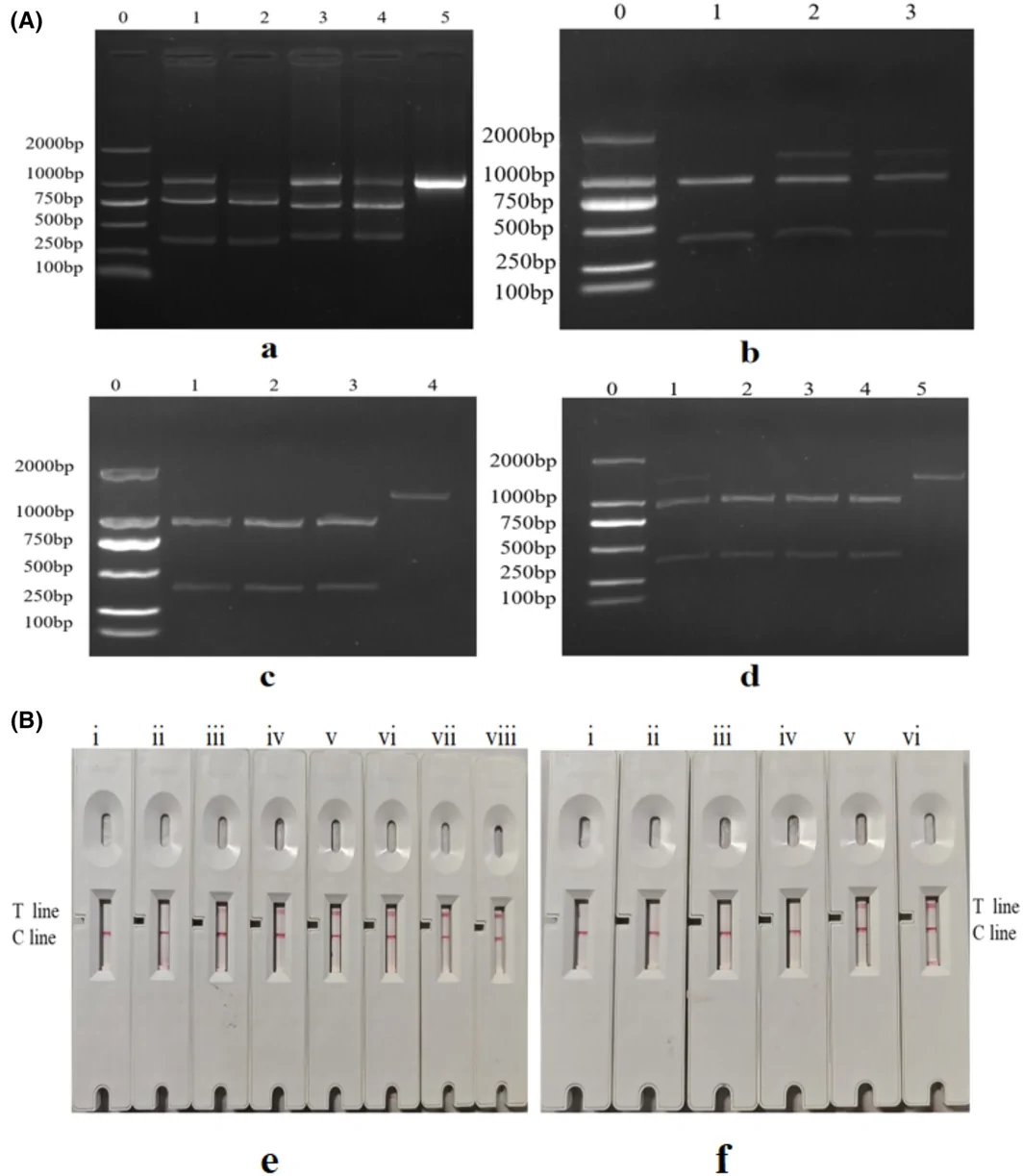
Optimization of CRISPR/Cas12a cleavage conditions and ssDNA reporter concentration.(A) Optimization of Cas12a‐mediated cis‐cleavage conditions. (a) Screening of candidate CrRNAs. (b) Optimization of reaction volume. (c) Optimization of the Cas12a: CrRNA molar ratio. (d) Optimization of incubation time. (B)Optimization of ssDNA reporter concentration for LFIA readout. (e) Baseline LFIA performance using intact BIO–FAM‐labeled ssDNA reporter at different concentrations. (f) Cas12a‐mediated trans‐cleavage of the ssDNA reporter at different reporter concentrations.

### Optimization of CRISPR–Cas12a cleavage parameters

The Cas12a cleavage conditions were optimized by evaluating reaction volume, Cas12a molar ratio, and incubation time using agarose gel electrophoresis (Fig. 3A). The disappearance of the full‐length substrate and the appearance of the expected cleavage fragments were used as the main criteria for assessing cleavage efficiency.

First, reaction volumes of 30, 50, and 100 μL were compared. As shown in Fig. 3A,B, complete cleavage was observed in the 30 μL reaction, whereas residual full‐length substrate was detected in the 50 and 100 μL reactions. Therefore, 30 μL was selected as the optimal reaction volume for subsequent experiments. Next, Cas12a molar ratios of 1:1, 1:2, and 1:3 were evaluated. All three ratios produced complete cleavage of the target fragment (Fig. 3A–C), indicating that increasing the amount of CrRNA did not further improve cleavage efficiency under the tested conditions. Considering reagent consumption and operational simplicity, a 1 : 1 Cas12a ratio was selected. Finally, incubation times of 5, 10, 20, and 30 min at 37 °C were tested. Incomplete cleavage was observed at 5 min, whereas complete cleavage was achieved after 10 min and remained comparable at 20 and 30 min (Fig. 3A–D). Thus, 10 min was selected as the optimal cleavage time.

Based on these results, the optimized CRISPR–Cas12a cleavage reaction was performed in a 30 μL system with a Cas12a molar ratio of 1 : 1 and incubation at 37 °C for 10 min. These conditions were used for subsequent LAMP product verification and LFIA‐based detection.

### Optimization of ssDNA reporter concentration

The ssDNA reporter concentration was further optimized to ensure clear LFIA interpretation and efficient Cas12a‐mediated trans‐cleavage (Fig. 3B). First, different concentrations of intact BIO–FAM‐labeled ssDNA reporter were applied to the LFIA strips to evaluate baseline strip performance. As shown in Fig. 3B‐a, clear deposition on both the T and C lines was observed when the reporter concentration was ≥ 0.5 μm, indicating that sufficient intact reporter was required to generate a visible negative pattern.

Next, ssDNA reporter concentrations of 0.5, 1, 5, 10, and 20 μm were tested in the CRISPR–Cas12a cleavage system. As shown in Fig. **3**B‐b, the reporter could be effectively cleaved at 0.5, 1, and 5 μm, whereas incomplete cleavage was observed at 10 and 20 μm, resulting in residual T‐line signals. Although 0.5 μm reporter was cleavable, the relatively weak T‐line signal in the absence of Cas12a activation could increase the risk of ambiguous interpretation. In contrast, excessive reporter concentrations may not be completely cleaved in positive reactions, potentially leading to false‐negative interpretation. Therefore, 1 μm was selected as the optimal ssDNA reporter concentration because it provided a clear negative LFIA pattern while allowing efficient cleavage in positive reactions.

### Validation of the optimized CRISPR–Cas cleavage system on LAMP products

After determining the optimal CRISPR–Cas system, we used LAMP products as the target genes for cleavage (Fig. 4). Due to the high cleavage activity of the CRISPR–Cas system, efficient cleavage was achieved even with large amounts of LAMP products. Agarose gel electrophoresis results showed an increase in the number of target region bands in LAMP products, indicating that the system not only recognizes and cleaves complex LAMP products but also breaks double strands within the target gene region, leading to an increase in the number of bands within the target region. This aligns with findings reported by Chen et al., where Cas12a protein cleaves double‐stranded DNA, generating staggered 5′ and 3′ ends at a pam‐distance, resulting in increased banding within the target gene region [42].

**Fig. 4:**
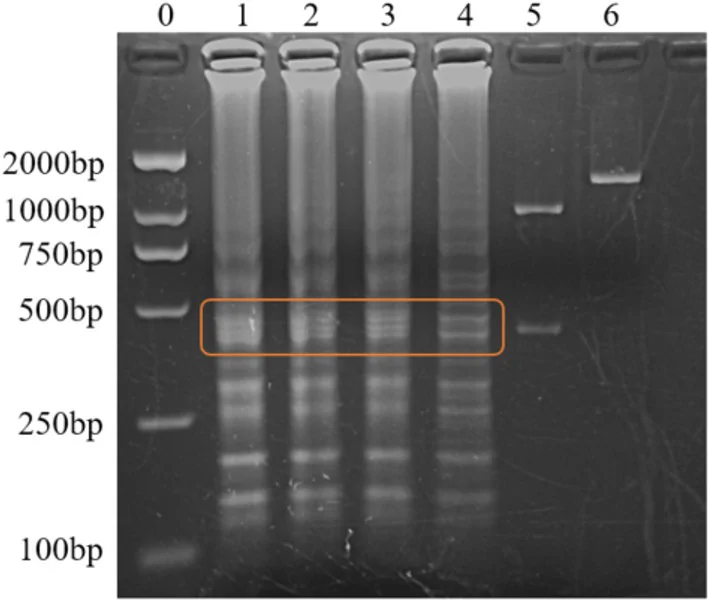
Verification of Cas12a‐mediated cleavage of LAMP products. Lane 1, LAMP product cleavage replicate 1; Lane 2, LAMP product cleavage replicate 2; Lane 3, LAMP product cleavage replicate 3; Lane 4, untreated LAMP product; Lane 5, digested dsDNA control; Lane 6, untreated dsDNA control.

### Sensitivity evaluation of LAMP–CRISPR/Cas12a with LFIA detection

To determine the LOD of this method, we employed *S. suis 2 (05ZYH33)* at concentrations ranging from 1.1 × 10^0^ CFU/mL to 1.1 × 10^7^ CFU/mL, with 10‐fold serial dilutions. As illustrated in Fig. 5, the LAMP reaction was successfully initiated at concentrations of 1.1 × 10^2^ CFU/mL and above. The inherent nature of LAMP technology enables exponential amplification of target DNA, even when present in minimal quantities within the reaction system. Consequently, the LAMP products generated from *S. suis 2 (05ZYH33)* at concentrations ≥ 1.1 × 10^2^ CFU/mL were effectively utilized in subsequent CRISPR–Cas cleavage reactions. Based on these experimental findings, we established the LOD of this method to be 1.1 × 10^2^ CFU/mL.

**Fig. 5:**
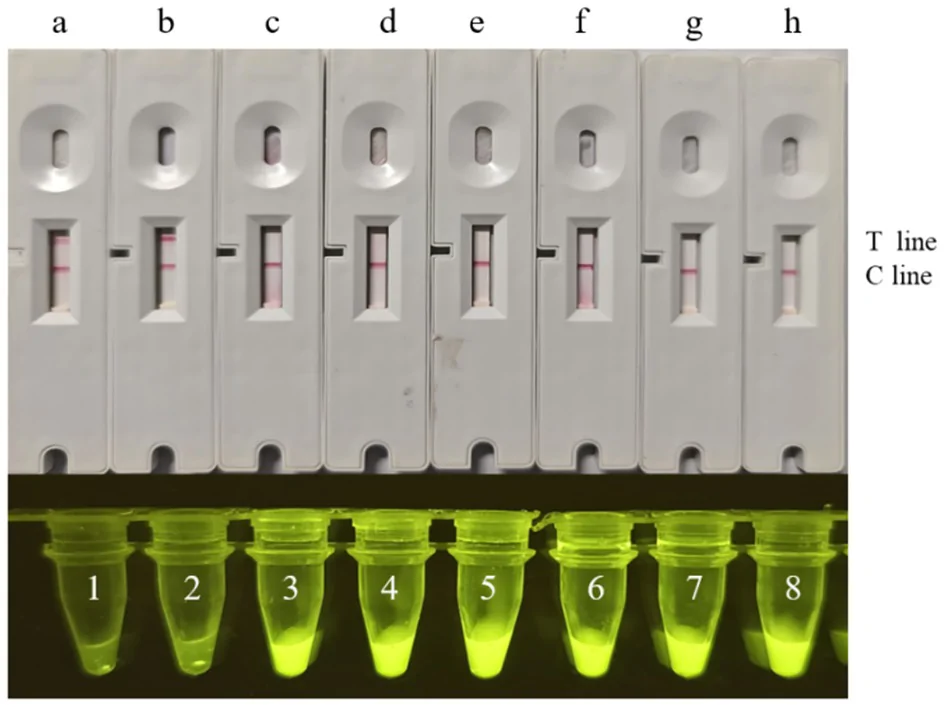
Determination of the limit of detection (LOD) of the LAMP–CRISPR/Cas12a–LFIA assay. *S. suis* 2 (05ZYH33) was subjected to serial 10‐fold dilutions, with bacterial concentrations in lanes A–H ranging from 1.1 × 10^0^ to 1.1 × 10^7^ CFU/mL.

### Specificity evaluation of the LAMP–CRISPR/Cas12a and LFIA detection platform

To assess analytical specificity, the proposed platform was evaluated using the *S. suis* strains and non‐*S. suis* strains listed in Table S1. As shown in Fig. 6A, the assay results were consistent with the expected distribution of the HtpsC target. Positive LFIA patterns were observed only for HtpsC‐positive *S. suis* strains, whereas HtpsC‐negative *S. suis* strains and all tested nontarget bacteria yielded negative results. Notably, *S. suis* 9 (22083), which lacks the HtpsC target, was not detected, consistent with the expected target specificity. These results demonstrate that the proposed assay exhibits high analytical specificity for HtpsC‐positive *S. suis* strains and shows no cross‐reactivity with the tested nontarget bacteria.

**Fig. 6:**
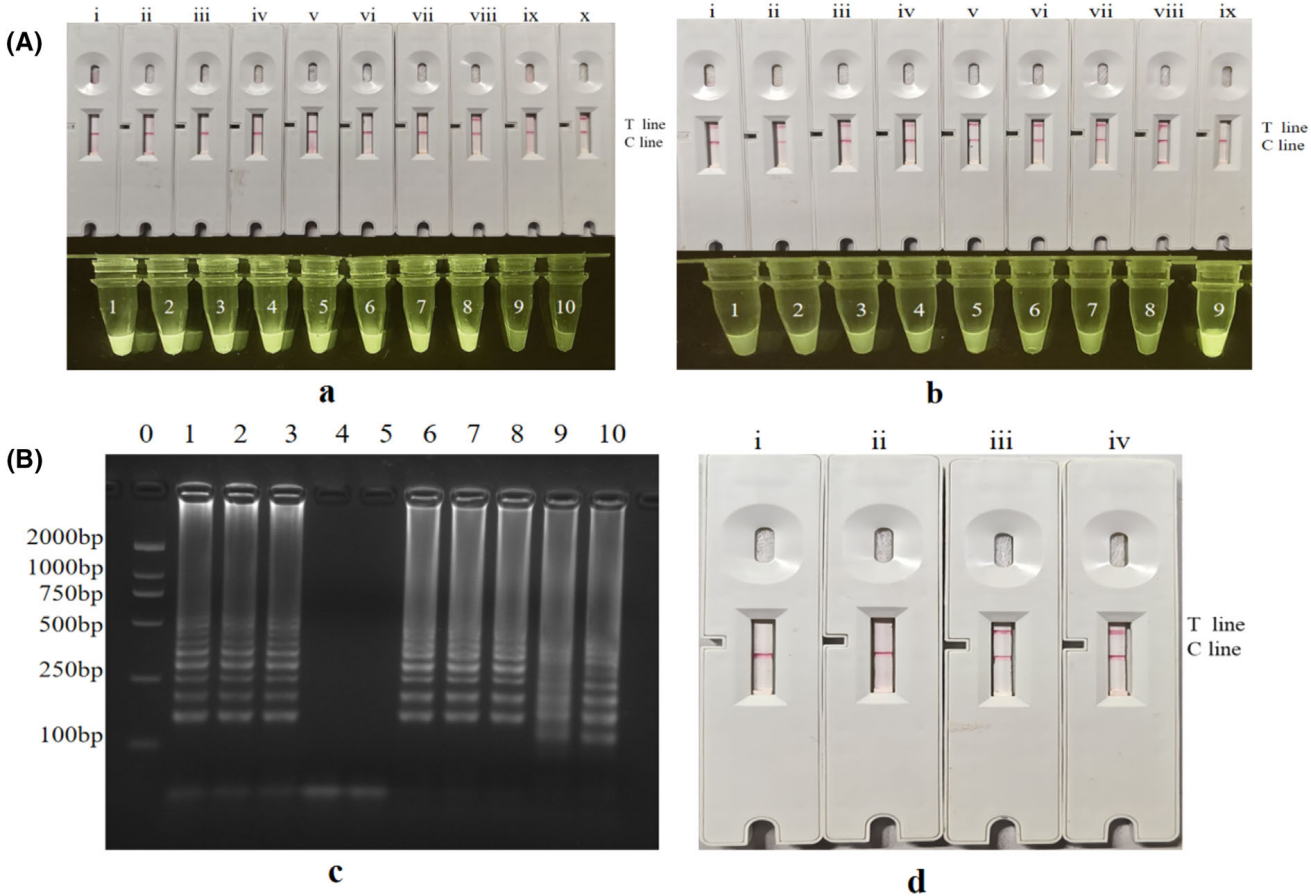
(A) Specificity validation of the LAMP–CRISPR/Cas12a–LFIA assay. (a) Intraspecies specificity analysis using different S. suis strains. (b) Interspecies specificity analysis using non‐S. suis bacterial strains. Detailed strain information is provided in Table S1. (B). Verification of CRISPR/Cas12a discrimination between true‐positive and false‐positive LAMP products. (c) Agarose gel electrophoresis of LAMP‐positive, negative, and false‐positive reactions. Lane 0: 2000‐bp marker; Lanes 1–3: LAMP‐positive products; Lanes 4–5: blank controls; Lanes 6–8: additional LAMP‐positive products; Lanes 9–10: false‐positive LAMP products. (d) LFIA verification results of representative positive, negative, and false‐positive products after CRISPR/Cas12a cleavage.

We further evaluated whether the CRISPR/Cas12a module could distinguish true target amplification from nonspecific LAMP products, thereby reducing false‐positive interpretation. In a set of parallel LAMP reactions, false‐positive amplification was observed in some reactions. The LAMP amplification results are shown in Fig. 6B(c). The LAMP products from Lanes 1 to 3, which showed positive amplification, Lanes 4 to 5, which were negative, Lanes 6 to 8, which were positive, and Lanes 9 to 10, which represented apparent false‐positive products, were further subjected to CRISPR/Cas12a cleavage and LFIA verification. The corresponding CRISPR/Cas12a–LFIA results are shown in Fig. 6B(d). True‐positive LAMP products activated Cas12a‐mediated trans‐cleavage and produced the expected positive LFIA pattern, characterized by suppression or disappearance of the T line with retention of the C line. In contrast, apparent false‐positive LAMP products failed to activate the CRISPR/Cas12a reaction and were ultimately interpreted as negative by LFIA. These findings indicate that the CRISPR/Cas12a module serves as a sequence‐specific verification step and can reduce false‐positive interpretation caused by nonspecific LAMP amplification.

### Detection of *S. suis 2 (05ZYH33)* in spiked blood samples

To further assess the performance of the proposed assay in a complex biological matrix, spiking experiments were performed using porcine blood samples (Fig. 7). *S. suis* 2 (05ZYH33) was artificially introduced into porcine blood at different concentrations and tested using the established LAMP–CRISPR/Cas12a–LFIA workflow. The assay detected spiked *S. suis* 2 (05ZYH33) in porcine blood under the tested conditions, and the nonspiked blood controls remained negative. The detection performance in spiked blood was consistent with that observed in artificially diluted bacterial suspensions, suggesting that porcine blood did not cause obvious matrix interference under the tested conditions. These results support the preliminary matrix compatibility of the assay, although further validation using real clinical samples is still required.

**Fig. 7:**
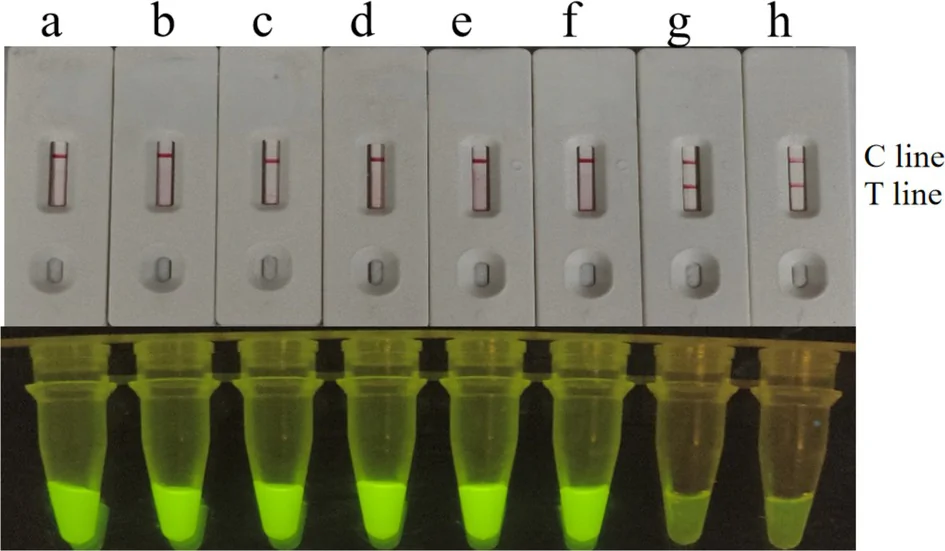
Detection of *S. suis* 2 (05ZYH33) in spiked porcine blood samples using the LAMP–CRISPR/Cas12a–LFIA assay. Panels a–h represent bacterial concentrations from 10^7^ to 10^0^ CFU/mL.

### Comparative validation of the LAMP–CRISPR/Cas12a–LFIA assay and conventional PCR for *Streptococcus suis* detection

To evaluate the performance of the developed LAMP–CRISPR/Cas12a–LFIA assay, the HtpsC‐targeted isothermal detection method was compared in parallel with conventional PCR. As shown in Table 1, key parameters, including sensitivity (Fig. S2), detection time, equipment requirements, and estimated cost, were systematically compared.

**Table 1:** Comparison of LAMP–CRISPR/Cas12a–LFIA and conventional PCR for *S. suis* detection.

Detection method	Sensitivity/LOD (CFU/mL)	Time (min)	Cost (US$/sample)	Equipment cost
PCR	10^3^	90	0.5	High
LAMP–CRISPR/Cas12a	1.1 × 10^2^	45	1.2	Low

## Discussion

HtpsC was selected as the molecular target in this study because of its relatively broad distribution and conservation among *Streptococcus suis* strains. Previous bioinformatic analysis showed that HtpsC is present in 119 of 151 sequenced *S. suis* genomes available in GenBank (78.8%), covering multiple serotypes [9]. In particular, HtpsC is widely distributed among several pathogenic serotypes, including Serotypes 1–9, 12, 16, and 23, supporting its value as a practical target for broad‐range detection of *S. suis*. Compared with conventional detection strategies based on capsular polysaccharide genes, HtpsC‐targeted detection may cover a wider range of *S. suis* serotypes [43, 44]. However, although most *S. suis* strains of different serotypes may harbor the HtpsC gene, strains belonging to the same serotype may either contain or lack HtpsC, as observed in Serotypes 4, 5, and 16. Therefore, this feature should be taken into account when interpreting negative results in epidemiological surveillance or diagnostic screening.

Among nucleic acid‐based methods for *Streptococcus suis* detection, PCR and qPCR are widely used because of their good specificity and sensitivity. Reported real‐time PCR assays can achieve sensitivities of < 10 copies per assay or 18.4 CFU [45]. However, these methods generally require thermal cyclers or real‐time fluorescence instruments, which limits their application in equipment‐limited or field settings. Isothermal amplification methods, such as LAMP and RPA, are more suitable for rapid detection because they do not require thermal cycling, and several reported *S. suis* LAMP‐ or RPA‐based assays have achieved rapid and sensitive detection. Nevertheless, amplification‐only assays may still be affected by nonspecific amplification or aerosol contamination. CRISPR‐based detection has strong sequence‐recognition capability and can effectively reduce false‐positive interpretation. Recently developed CRISPR‐coupled nucleic acid detection platforms have further improved the specificity of isothermal amplification‐based assays [46, 47, 48]. In 2024, Wang et al. developed an RPA–CRISPR/Cas12a platform for the detection and serotyping of *S. suis* Serotype 2, achieving a detection limit of 10 CFU within less than 60 min [49]; however, this method was mainly designed for *S. suis* Serotype 2 detection. In 2025, Yang et al. established an RAA–CRISPR/Cas12a assay targeting the recN gene for *S. suis* detection, with reported sensitivities of 2.44 × 10^1^ copies/μL and 2.1 × 10^1^ CFU within 30 min [1]. However, because it relies on fluorescence readout, its suitability for field use is limited, which restricts its application at primary‐level facilities. In the present study, we established an HtpsC‐targeted LAMP–CRISPR/Cas12a platform and incorporated lateral flow immunochromatography to enable visual readout of the reaction results. The assay showed 100% specificity for HtpsC‐positive *S. suis* strains in the tested panel and showed no cross‐reactivity with closely related or common nontarget bacteria, including *Streptococcus agalactiae, Enterococcus faecalis, Streptococcus pyogenes, Staphylococcus aureus, and Escherichia coli*. The detection limit of the assay for pure cultures of *S. suis* was 1.1 × 10^2^ CFU/mL. Due to limitations in obtaining clinical specimens, spiked porcine blood samples were used for preliminary matrix evaluation. The results showed that the assay maintained a detection limit of 10^2^ CFU/mL in blood‐spiked samples, indicating that the blood matrix did not cause significant interference under the test conditions and suggesting its potential application for clinical sample detection. Moreover, the method does not rely on fluorescence readers and can be visually interpreted using lateral flow strips, indicating its potential for on‐site detection of practical samples. Compared with conventional PCR, the estimated cost per test of the present assay is higher, approximately US$1.2, compared with US$0.5 for PCR. However, this additional cost can be justified by its shorter detection time, portable workflow, reliable visual readout, and reduced dependence on specialized laboratory equipment in field settings.

Another important advantage of this platform is its ability to reduce the false‐positive interpretations commonly associated with LAMP assays. Although LAMP is rapid and highly efficient, it is susceptible to aerosol contamination, particularly in laboratories where LAMP is performed frequently. In this study, the false‐positive LAMP products observed in repeated experiments failed to activate the CRISPR/Cas12a trans‐cleavage reaction and were ultimately interpreted as negative in the LFIA readout. These findings indicate that the CRISPR/Cas12a step serves as a sequence‐specific verification layer, enabling discrimination between true target‐derived amplicons and nonspecific LAMP products. This thereby improves the specificity of the assay and reduces the risk of result misinterpretation.

Nevertheless, several limitations should be acknowledged. First, although HtpsC is present in most *S. suis* strains, some strains may lack this gene and thus escape detection. Therefore, evaluation of alternative or multiple molecular targets may further improve strain coverage and assay robustness. Second, although the applicability of the assay in a complex biological matrix was preliminarily evaluated using spiked porcine blood samples, further validation using naturally infected clinical specimens is required before the diagnostic performance can be conclusively established. Third, although the present study included a conventional PCR comparison with the proposed method, an independent qPCR analysis should be performed in future work to provide a more direct and quantitative assessment of detection performance. Fourth, although we have developed a visual assay, we still consider it necessary to incorporate quantitative T/C line analysis using imagej or a dedicated strip reader in future studies, as this would be highly valuable for the next‐stage development of this method. Finally, a one‐step procedure could be explored to reduce contamination risk and improve the assay's suitability for field applications.

## Conclusion

This study developed a rapid and visual detection method targeting the HtpsC gene by integrating LAMP, CRISPR/Cas12a‐mediated sequence verification, and lateral flow immunochromatography. The results indicate that the established platform has good analytical sensitivity and specificity, reduces reliance on specialized instruments, and shows potential for rapid visual screening of Streptococcus suis. Further validation using naturally infected samples and more comprehensive comparative evaluation will be required before broader diagnostic or field application.

## Conflict of interest

The authors declare no conflicts of interest.

## Author contributions

MC and YL conceived and designed the project, and were responsible for methodology, conceptualization, funding acquisition, resources, and supervision; they also contributed to writing—review and editing. LL acquired the data. LL, MX, XC, SY, and FM performed the data curation, analyzed and interpreted the data, and wrote the original draft. XX contributed to the resources, validation, and writing—review and editing.

## Funding

The authors declare that they received funding support during the research, writing, and/or publication of this article. This work was supported by the National Natural Science Foundation of China (grant nos.: 81472932 and 32572888).

## Supporting information


**Table S1.** Bacterial strains used in this study.
**Table S2.** Relevant sequences used in this study.
**Fig S1.** Four CrRNA sequences designed within the HtpsC target gene region. In the experiment optimizing CrRNA sequences, we designed four CrRNAs within the target gene region of the LAMP reaction through PAM recognition sites, and verified the cleavage efficiency of the four CrRNAs using the cis‐cleavage capability of the Cas12a protein. As shown in the figure, these are the sites of the four CrRNAs within the target region.
**Fig S2.** One percent agarose gel electrophoresis image of conventional PCR products from pure‐culture *S. suis* 2 (05ZYH33) suspensions. Panels a–h: bacterial concentrations from 10^7^ to 10^1^ CFU/mL.

## Data Availability

All data of this article are available from the corresponding author upon request.
