# Supplementary material for: Ultrafast visual detection of Streptococcus suis via HtpsC‐targeted LAMP–CRISPR–Cas12a coupled with lateral flow immunochromatography
Source: FEBS Open Bio. 2026 Oct 16:10.1002/2211-5463.70353. Online ahead of print. doi: 10.1002/2211-5463.70353 (PMC13673931; doi:10.1002/2211-5463.70353)
Supplement: Supplementary file 1 [file FEB4-9999-0-s001.pdf]

Supporting Information

Table S1 Bacterial strains used in this study.
Table S2 Relevant sequences used in this study.
Figure S1 Four CrRNA sequences designed within the HtpsC target gene region
Figure S2 1% agarose gel electrophoresis image of conventional PCR products from pure-culture *S. suis* 2 (05ZYH33) suspensions.

Table S 1

Bacterial strains used in this study.

Strain	Laboratory name	Serotype	Source/ Reference	Culture medium	Growing temperature
S.suis	05ZYH33	2	Prof.Marecelo Gottschalk	THB Broth	37°C
	WZ48-1	2	Prof.Ming Li	THB Broth	37°C
	T15	2	Prof.Astrid de Greeff	THB Broth	37°C
	NCTC 10234	2	Prof.Marecelo Gottschalk	THB Broth	37°C
	5,428	1	Prof.Marecelo Gottschalk	THB Broth	37°C
	2,524	4	Prof.Marecelo Gottschalk	THB Broth	37°C
	8,074	7	Prof.Marecelo Gottschalk	THB Broth	37°C
	22,083	9	Prof.Marecelo Gottschalk	THB Broth	37°C
	13,730	14	Prof.Marecelo Gottschalk	THB Broth	37°C
S.aureus	ATCC 29213		GDMCC	LB Broth	37°C
S.pyogenes	ATCC 19615		GDMCC	Brain Heart Infusion Broth	37°C
S.agalactiae	GDMCC 1408		GDMCC	Brain Heart Infusion Broth	37°C
S.enteriditis	ATCC 14028		GDMCC	LB Broth	37°C
E.coli	GDMCC 801268		GDMCC	LB Broth	37°C
E.faecalis	ATCC 51299		GDMCC	Brain Heart Infusion Broth	37°C
L.monocytogenes	ATCC 54004		GDMCC	Listeria Enrichment Broth Base	37°C

Table S 2

Relevant sequences used in this study.

Name	Prime 5’-3’
HtpsC-F	TCTAAATATTGGTCAGCGAAAAGATCCGC
HtpsC-R	TCTTAGGCACTAGGTCTTTACCAAACATGAG
F3	TGAGGGACTGGTCTTAGAACAT
B3	TGATCTCCATGACGTACCGTA
FIP	TGGAATGCGAGCTGGATTGGCTTTTAAATGGTCCTATCTGGTACCCA
BIP	GCAAAGCAAGCGAAGCTATCACAATTTTCATTCGCATCTTCAGCCACTA
CrRNA-1	UAAUUUCUACUCUUGUAGAUAUGAUGGUUAUGUAUUUGAU
CrRNA-2	UAAUUUCUACUCUUGUAGAUUGAUAGCUUCGCUUGCUUUG
CrRNA-3	UAAUUUCUACUCUUGUAGAUCGUUGGAAUGCGAGCUGGAU
CrRNA-4	UAAUUUCUACUCUUGUAGAUUCGGUGAUGUAGGAAGCCUA
ssDNA	BIO-TTTTTATTTTT-FAM

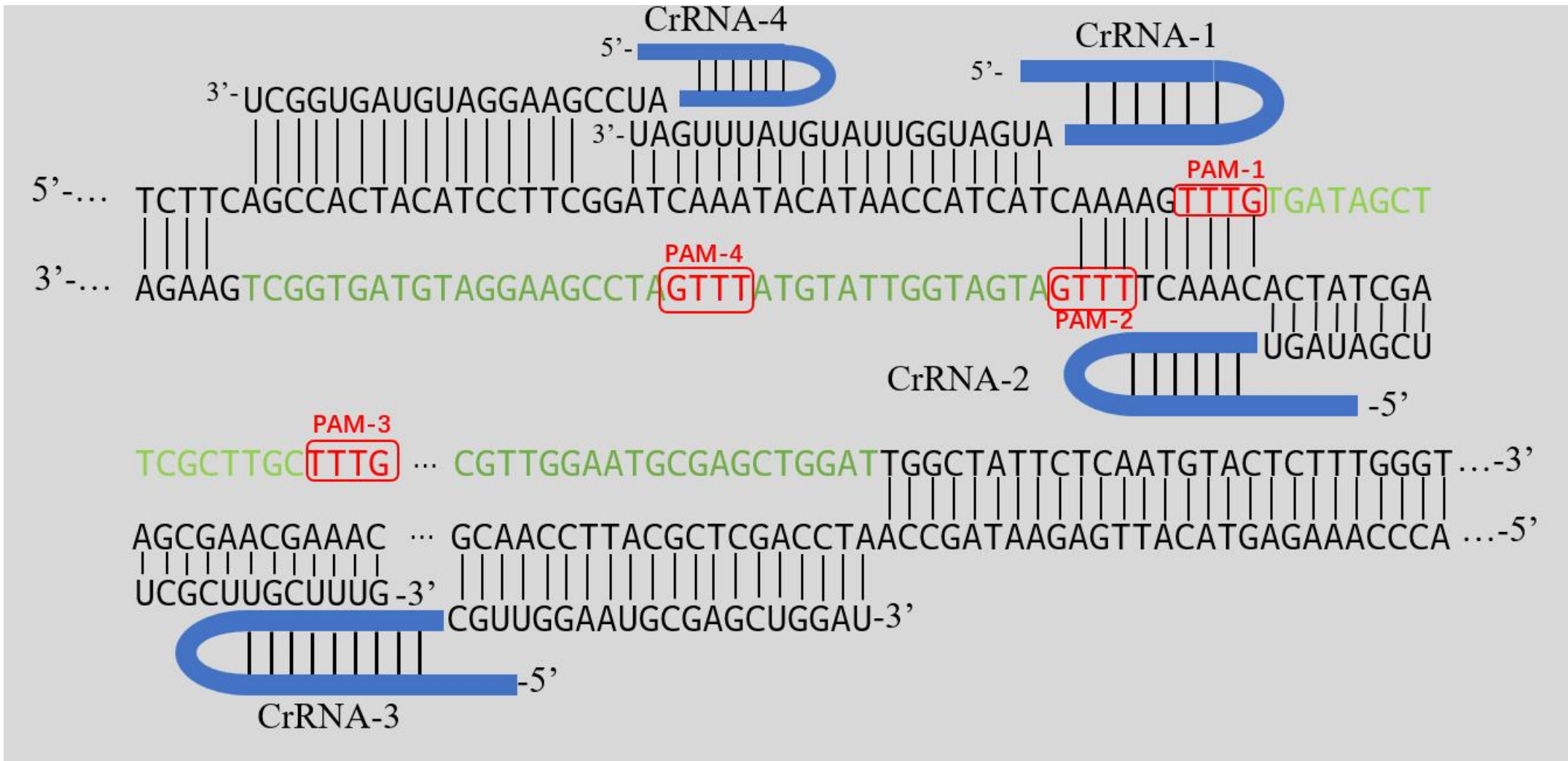

Figure S1

Four CrRNA sequences designed within the HtpsC target gene region

In the experiment optimizing CrRNA sequences, we designed four CrRNAs within the target gene region of the LAMP reaction through PAM recognition sites, and verified the cleavage efficiency of the four CrRNAs using the cis-cleavage capability of the Cas12a protein. As shown in the figure, these are the sites of the four CrRNAs within the target region.

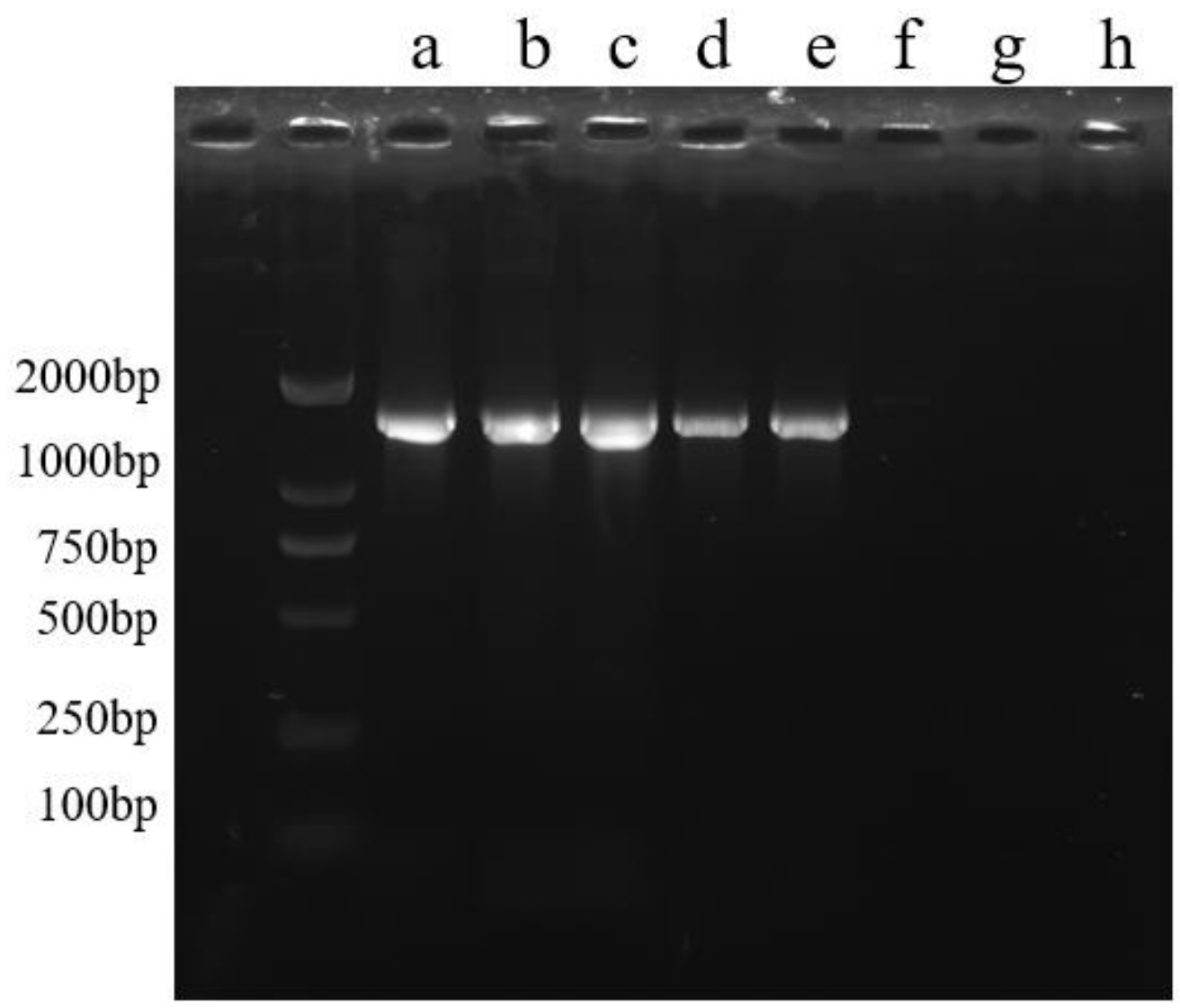

Figure S2:1% agarose gel electrophoresis image of conventional PCR products from pure-culture *S. suis* 2 (05ZYH33) suspensions. Panels a–h: bacterial concentrations from 10^7 to 10^1 CFU/mL.
